# Comparison of continuous versus intermittent enteral nutrition in critically ill patients (COINN): study protocol for a randomized comparative effectiveness trial

**DOI:** 10.1186/s13063-020-04866-2

**Published:** 2020-11-23

**Authors:** Ondrej Hrdy, Kamil Vrbica, Eva Strazevska, Petr Suk, Lenka Souckova, Radka Stepanova, Igor Sas, Roman Gal

**Affiliations:** 1 Department of Anesthesiology and Intensive Care Medicine, University Hospital Brno and Faculty of Medicine, Masaryk University, Brno, Czech Republic; 2 Department of Pharmacology, Faculty of Medicine, Masaryk University, VVI CZECRIN, Brno, Czech Republic

**Keywords:** Critical care, Enteral nutrition, Intensive care unit, Gastric residual volume, Diarrhea

## Abstract

**Background:**

Enteral nutrition is part of the treatment of critically ill patients. Administration of enteral nutrition may be associated with signs of intolerance, such as high gastric residual volumes, diarrhea, and vomiting. Clinical trials regarding the effects of the mode of administration of enteral nutrition on the occurrence of these complications have yielded conflicting results. This trial aims to investigate whether the mode of administration of enteral nutrition affects the time to reach nutritional targets, intolerance, and complications.

**Methods:**

COINN is a randomized, monocentric study for critically ill adult patients receiving enteral nutrition. Patients will be randomly assigned to two groups receiving (1) continuous or (2) intermittent administration of enteral nutrition. Enhancement of enteral nutrition will depend on signs of tolerance, mainly the gastric residual volume. The primary outcome will be the time to reach the energetic target. Secondary outcomes will be the time to reach the protein target, tolerance, complications, hospital and ICU lengths of stay, and 28-day mortality.

**Discussion:**

This trial aims to evaluate whether the mode of application of enteral nutrition affects the time to reach nutritional targets, signs of intolerance, and complications.

**Trial registration:**

ClinicalTrials.gov NCT03573453. Registered on 29 June 2018

## Administrative information

**Table:** 

Title {1}	Comparison of Continuous Versus Intermittent Enteral Nutrition in Critically Ill Patients (COINN): study protocol for a randomized comparative effectiveness trial.
Trial registration {2a and 2b}.	ClinicalTrials.gov: NCT03573453
Protocol version {3}	Protocol version 2.0, dated May 28th, 2019.
Funding {4}	This study is not funded.
Author details {5a}	OH^1^, KV^1^, ES^1^, PS^1^, LS^2^, RS^2^, IS^1^, RG^1^ ^1^ Department of Anesthesiology and Intensive Care Medicine, University Hospital Brno and Faculty of Medicine, Masaryk University, Brno, Czech Republic. ^2^ Department of Pharmacology, Faculty of Medicine, Masaryk University, VVI CZECRIN
Name and contact information for the trial sponsor {5b}	**Trial Sponsor**: Masaryk University Brno **Contact name**: prof. MUDr. Martin Smrcka, Ph.D., MBA **Address**: Jihlavska 20, 625 00 Brno **Telephone**: +420532232884 **Email**: smrcka.martin@fnbrno.cz
Role of sponsor {5c}	The sponsor has no role in the design of this trial and will not have any role during its execution, analyses, interpretation of the data, or the decision to submit results.

## Introduction

### Background and rationale {6a}

Gastric enteral nutrition (EN) is commonly used in critically ill patients who cannot be fed orally [1, 2]. It can be performed with a continuous infusion or the administration of several boluses via a gastric tube. Continuous feeding consists of nutritional infusion at a constant rate. Intermittent nutrition involves administration of a bolus volume delivered over 15–40 min multiple times per day [3]. The rate of continuous infusion or the volume of the bolus is set to meet the daily energetic target. EN begins with the administration of a small amount of nutrition. If the EN is tolerated well, the amount of administered nutrition is increased until the target energy is reached. When signs of intolerance are recognized, the amount of administered nutrition is decreased; thus, the tolerance toward EN determines the time to reach the estimated energetic target—the primary outcome of this study. Both methods have advantages and disadvantages. Continuous administration of nutrients is unphysiologic, does not promote anabolism, and is associated with metabolic complications [4]. However, continuous administration is also associated with fewer gastrointestinal or respiratory complications, and meeting the nutritional targets earlier [5]. Intermittent feeding is physiologic and can promote proteosynthesis. However, it leads to high gastric residual volumes (GRVs) and higher occurrence of diarrhea and aspiration [6]. Studies investigating the effects of the mode of administration on the occurrence of complications and other outcomes have yielded conflicting results [6–9]. The authors of a systematic review of the effectiveness of continuous versus intermittent enteral nutrition have been unable to affirm recommendations for one method over the other [10]. The new guidelines of the European Society for Parenteral and Enteral Nutrition (ESPEN) on clinical nutrition in intensive care units recommend the use of continuous rather than bolus EN. However, the authors assumed that evidence on this issue is very limited, that bolus and continuous feeding are similar in achievement of nutritional targets without either increasing the rate of adverse effects, and that bolus feeding may be a greater stimulus for protein synthesis [11]. On the basis of current knowledge, a clear recommendation for a preferred method of administration of enteral feeding cannot be made [12, 13]. The aim of this study is to determine whether any difference exists in the time to reach the energetic target between two tolerance-driven modes of administration of EN in critically ill adult patients.

## Objectives {7}

The primary study objective is to compare continuous and intermittent modes of application of enteral feeding on the time to reach an energetic target in critically ill adult patients.

Key secondary objectives are to determine the effects of the mode of administration of enteral feeding on the time to reach the protein target, the gastrointestinal tolerance (on the basis of assessment of GRV), vomiting, diarrhea, and the need for postpyloric nutrition. Other secondary objectives are complications of enteral feeding, as assessed by the incidence of aspiration and ventilator-associated pneumonia (VAP).

## Trial design {8}

The COINN trial is designed as a prospective monocentric explorative trial with two parallel arms. The primary endpoint is the time to reach the energetic target in critically ill adult patients on enteral feeding. Block randomization with a 1:1 allocation will be performed.

## Methods: participants, interventions, and outcomes

### Study setting {9}

The COINN trial is currently ongoing in four intensive care units (ICUs) at one academic hospital in the Czech Republic.

### Eligibility criteria {10}

All patients admitted to the ICU are screened for the inclusion criteria. After informed consent is obtained, each patient is randomized to one of the trial arms. The randomization occurs before EN is started.

The inclusion criteria are as follows:

1. Age 18–80 years
2. Indication of EN with a nasogastric or an orogastric tube
3. Nutrition Risk in the Critically Ill (mNUTRIC) score ≥ 5
4. Body mass index 18–50
5. Mechanical ventilation expected for at least 72 h

Patients meeting any of the following criteria are excluded:

1. Upper gastrointestinal tract surgery in previous medical history
2. Bowel obstruction
3. Bowel ischemia
4. Acute pancreatitis
5. Severe diarrhea (> 1 L/24 h)
6. Gastrointestinal bleeding
7. Short bowel syndrome
8. Malabsorption syndrome in previous medical history
9. Ongoing EN on admission to ICU

### Who will take informed consent? {26a}

A trained study investigator will describe the study to patients or to authorized surrogates if applicable. Patients (or authorized surrogates) will also receive information sheets. The study investigator will discuss the study with patients (or authorized surrogates) in light of the information provided in the information sheets. The study investigator will obtain written consent from patients (or authorized surrogates) willing to participate in the trial. In the case of patients’ inability to consent because of a medical condition, the ability to participate in the trial will be assessed by a medical council consisting of one independent physician informed about the study details and one study investigator.

### Additional consent provisions for collection and use of participant data and biological specimens {26b}

Not applicable. Ancillary studies using participant data and biological specimens are not planned.

## Interventions

### Explanation for the choice of comparators {6b}

EN can be administered as separate bolus doses several times per day or as a continuous infusion over a long period of time. Continuous administration is favored by current ESPEN guidelines and will serve as the comparator for the intervention of intermittent feeding.

### Intervention description {11a}

The position of the gastric tube will be verified by measurement of gastric pH or abdominal X-ray. Commercial isocaloric formula with a high content of whey-based protein will be used. To eliminate the effects of formula composition on primary and secondary outcomes of the study, the same enteral formula will be used in all patients included in both study arms. Energetic and protein targets will be estimated according to current guidelines, on the basis of body mass index (BMI) and actual body weight (ABW), as summarized in Table 1 [1]. The energy and protein targets will be determined by the study investigator. The study investigator will provide information to the nurse and attending physician regarding the arm of the study to which the patient was assigned, the time of initiation of EN, and the target rate and bolus volume.

Administration of EN begins no earlier than 6 am and no later than 6 pm if signs of intolerance are absent (see below) and the GRV is below 500 mL. Otherwise, the start of EN will be postponed to the time of the next GRV measurement.

EN will be administered in the first arm (CONT) with continuous infusion via an enteral pump with an infusion time of 18 h per day. During the infusion, the patient will be in a 30–40° head-up position. The initial rate of infusion will be set to 25 mL/h and gradually increased every 4 h according to clinical signs of intolerance and GRV measurements. Signs of intolerance will be considered if abdominal distension or pain, vomiting, or diarrhea occur, and if the GRV exceeds 500 mL.

The rate of infusion will be increased according to tolerance and GRV. If there is no sign of intolerance, and the GRV is < 250 mL in two consecutive measurements, the rate will be increased by 25 mL/h until the target rate is reached. If signs of intolerance are absent, and the GRV is 250–500 mL, the current rate will remain unchanged. If intolerance signs are observed, or the GRV is > 500 mL, the rate will be decreased to half until the next assessment of tolerance and GRV.

The daily dose of EN will be divided in the second arm (INT) into six portions administered in 4-h intervals via an enteral pump. The rate of the enteral pump will depend on the volume of the bolus. To avoid sudden gastric distension, the rate will increase with increasing bolus volume [4]. The rates of the enteral pump for particular volumes of EN are summarized in Table 2.

During the infusion of EN, the patient will be in a 30–40° head-up position. After the administration is finished, the gastric tube will be rinsed with 20 mL of water. The initial volume of the bolus will be 80 mL and will be gradually increased every 4 h according to clinical signs of intolerance and GRV measurements. Signs of intolerance will be considered as abdominal distension or pain, vomiting, diarrhea, and a GRV exceeding 500 mL.

The rate of infusion will be increased according to tolerance and GRV. If there is no sign of intolerance and the GRV is < 250 mL in two consecutive measurements, the volume will be increased by 80 mL/h until the target volume is reached. If intolerance signs are absent and the GRV is 250–500 mL, the current volume of the bolus will remain unchanged. If intolerance signs or a GRV > 500 mL is observed, the volume will be decreased to half until the next assessment of tolerance and GRV.

Measurement of GRV will be performed throughout the entire study period (days 1–5) in 4-h intervals with a 150-mL syringe used for aspiration of gastric content. Volumes lower than 150 mL will be returned to the stomach, and larger amounts will be discarded.

**Table 1:** Energy and protein targets

BMI	Energy	BMI	Protein
< 30	25–30 kcal/kg of ABW/24 h	< 30	1.2–2 g/kg ABW/24 h
30–50	11–14 kcal/kg of ABW/24 h	30–40	2 g/kg ABW/24 h
–	–	> 40	2–2.5 g/kg ABW/24 h

### Criteria for discontinuing or modifying allocated interventions {11b}

Not applicable. Modification and discontinuation of study interventions is described in the previous section.

### Strategies to improve adherence to interventions {11c}

Administration of EN will be provided by nurses who will receive training before the study begins. During the training, the principal investigator or another responsible member of the study team will present the objectives of the study, the methods of measuring patient height and weight, the protocol of administration of EN in both study arms, the method of measuring GRV, and how other study variables will be assessed and documented in the patient medical records. After the study commencement, re-training will occur every 6 months until the trial ends.

### Relevant concomitant care permitted or prohibited during the trial {11d}

#### Prokinetics of the upper gastrointestinal tract

Prokinetics will be administered if an increase in the rate or bolus volume is not reached in three consecutive assessments of tolerance and GRV measurements, respectively. If the rate or bolus volume is lowered in two consecutive assessments of tolerance and GRV measurements, the administration of prokinetics will also start. The choice and dose of prokinetic agent will be made by the attending physician. The effects of prokinetics will be assessed after 24 h of administration. If intolerance persists and the GRV is below 250 mL, EN will continue on a fixed rate of 25 mL/h in both arms. If intolerance and GRV > 250 mL persist, a postpyloric tube will be inserted, and postpyloric nutrition will commence.

**Table 2:** Volumes of boluses and rates of the enteral pump

Bolus volume (mL)	Rate of enteral pump (mL/h)	Time of administration (min)
80	160	30
160	320	30
240	320	45
320	320	60
400	400	60
> 400	400	Until administered

#### Parenteral nutrition

The administration of parenteral nutrition will be associated with institutional standards of care. Parenteral nutrition may be considered if less than 60% of the nutritional goal on days 7–10 is reached.

#### Prokinetics of the lower gastrointestinal tract

The prokinetics of the lower gastrointestinal tract will be indicated by the attending physician any time during the study period in the absence of signs of bowel movements. The choice of agent, dosage, and route of administration will be made by the attending physician.

### Provisions for post-trial care {30}

Not applicable.

The trial period includes the first 5–7 days of critical illness. Administration of EN in this trial will follow current guidelines [1, 11], and participation in the trial carries no additional risks beyond those arising from the critical illness itself.

### Outcomes {12}

The primary outcome of the trial is the time from the beginning of the administration of EN to the time at which at least 80% of the target energy is delivered. This will be reported as the day of EN administration. The Kaplan-Meier time-to-event analysis will be used for the analysis of the primary outcome.

Secondary outcomes:

1. Time from the beginning of the administration of EN to the time at which at least 80% of the target protein dose is delivered. This will be reported as the day of EN administration. The Kaplan-Meier time-to-event method of analysis will be used for the analysis of this secondary outcome.
2. Achievement of the energetic target. This outcome will be aggregated as the proportion of patients who achieve the energetic target during the study period.
3. Tolerance of EN GRV will be aggregated as the proportion with GRV < 250 mL, 250–500 mL, and > 500 mL. Episodes of vomiting will be aggregated as the proportion of patients with any number of episodes of vomiting. Diarrhea stools will be aggregated as the proportion of patients with diarrhea stool > 1 L/day. The need for postpyloric nutrition will be aggregated as the proportion of patients with this need.
4. Incidence of complications of EN. The number of aspiration episodes will be aggregated as the proportion of patients with any number of episodes of aspiration. VAP will be aggregated as the proportion of patients with confirmed VAP.
5. Total serum protein, albumin, and prealbumin concentrations on days 1 and 5. Serum concentrations of protein, albumin, and prealbumin will be aggregated as the mean.
6. ICU and hospital length of stay. ICU and hospital length of stay will be aggregated as the mean. The day of admission and day of discharge will be counted as 1 day.
7. The 28-day mortality will be evaluated with a Kaplan-Meier curve.

### Participant timeline {13}

The schedule of enrolment, interventions, and visits is summarized in Table 3.

### Sample size {14}

Studies focused on the primary target of our study are lacking. However, we have identified several studies comparing bolus and continuous administration of enteral feeding. These studies included small numbers of patients, were conducted in different patient populations, and did not assess the time to reach the nutritional targets as a primary outcome. However, for calculation of the sample size, we hypothesized a difference of 1 day between groups (target being reached on day 2 and day 3, respectively). The alpha was set to 5%, and the power was set to 80%. The estimated sample size was 122 patients per arm. To account for potential drop-out, we increased the sample size to 300 participants in total, with 150 patients in each study arm.

**Table 3:** Participant timeline

	Study period							Follow-up
Time point	Screening	D0	D1	D2	D3	D4	D5	D28
Number of visits	1	2	3	4	5	6	7	8
Eligibility criteria	x							
Informed consent	x							
Allocation	x							
Demographic data		x						
APACHE II		x						
SOFA score		x						
Laboratory tests (total serum protein, albumin, prealbumin)		x					x	
Estimation of energy and protein target		x						
Administration of enteral nutrition			x	x	x	x	x	
Assessment of tolerance (diarrhea, vomiting, GRV, postpyloric nutrition)			x	x	x	x	x	
Assessment of complications			x	x	x	x	x	
Assessment of energetic and protein targets			x	x	x	x	x	
Assessment of hospital LOS, ICU LOS, and mortality								x

### Recruitment {15}

Recruitment of patients began in November 2018. During the first 12 months of the study, 787 patients were screened, among whom 100 were allocated to one of the two study arms. Allocation of the last patient is anticipated to occur in November 2021.

## Assignment of interventions: allocation

### Sequence generation {16a}

COINN is a single-center study. The randomization procedure is implemented in an electronic case report form (eCRF) with a randomization ratio of 1:1. The randomization list was generated by the study statistician and implemented in the eCRF by the study data manager.

### Concealment mechanism {16b}

The web-based randomization system ensures allocation concealment.

### Implementation {16c}

The allocation sequence generation is embedded in the trial web site. The study investigator enrolls participants and randomizes them by using the web-based randomization system.

## Assignment of interventions: blinding

### Who will be blinded {17a}

The study staff members and treating physicians and other staff will not be blinded, because of the impossibility of blinding while performing the study intervention. The data management group and statisticians will work with a pseudonymized data set.

### Procedure for unblinding if needed {17b}

Not applicable.

This trial is not blinded.

## Data collection and management

### Plans for assessment and collection of outcomes {18a}

#### Demographic and baseline data

The study will collect demographic and baseline characteristics from medical records and electronic medical records, including age, sex, type of admission, and baseline characteristics. Patient weight will be measured on admission in a standard manner by using a bed-integrated scale; patient height will be measured on admission through a standardized protocol with a tape measure. The patient will lie flat in a supine position. A tape measure will be placed perpendicular to the patient, and the distance from the patient’s heels to the head will be measured.

Conventional scoring systems validated for critically ill patients will be used. Estimation of APACHE II, SOFA, and mNUTRIC scores will be performed by the attending physician and documented in the medical records. Results of laboratory tests will be documented in the electronic medical records and entered by the trial investigator in the eCRF.

#### Primary outcome

The time to reach the energetic target will be defined as the number of days from the beginning of the administration of EN until the day at which at least 80% of the planned EN is administered. The trial investigator will estimate the amount of EN administered by analyzing medical records in which the volume of EN is documented according to institutional standards.

#### Secondary outcomes

The time to reach the protein target will be defined as the number of days from the beginning of the administration of EN to the day at which at least 80% of the planned protein administration has occurred. The trial investigator will estimate that day by analyzing the medical records.

GRVs, episodes of vomiting, the number and volume of diarrhea stools, and the need for postpyloric nutrition will be documented in the medical records and entered in the eCRF by the trial investigator. Diarrhea will be defined as passing of loose stools three or more times per day. The number and volume of stools will be documented in the medical records. Volumes of diarrhea stools exceeding 1 L/day will be reported.

Aspiration of EN is defined as the presence of EN in the airway of the patient. It will be assessed by a nurse during standard airway care and documented in the medical records. The number of aspiration episodes and the incidence of VAP will be entered in the eCRF by the study investigator. Total serum protein, albumin, and prealbumin concentrations on days 1 and 5 will be obtained from the electronic medical records and entered in the eCRF by the study investigator. The ICU and hospital length of stay will be entered in the eCRF by the study investigator after analysis of the medical records. The information on whether the patient is alive or dead on day 28 will be obtained by telephone.

### Plans to promote participant retention and complete follow-up {18b}

After a participant is enrolled or randomized, the study site will make every reasonable effort to follow the participant for the entire study period. Study site staff members will be responsible for developing and implementing local standard operating procedures to achieve maximal follow-up.

### Data management {19}

The study data will be entered online in an Internet-based database and collected from medical records. The study staff members will have access to the medical records of the patients. Investigators will be responsible for (1) screening of patients, (2) obtaining informed consent, (3) randomization, and (4) collecting study data and entering the data in the eCRF. The statistician will analyze the study data in cooperation with the principal investigator. The data will be stored for 15 years after completion of the study and then destroyed.

To promote data quality, the eCRFs of each participant will be reviewed by another member of the study team.

### Confidentiality {27}

All study-related information will be stored securely at the study site. All participant information will be stored in locked file cabinets in areas with limited access. All local databases will be secured with password-protected access systems. Forms, lists, logbooks, appointment books, and any other listings that link participant ID numbers to additional identifying information will be stored in a separate locked file in an area with limited access.

### Plans for collection, laboratory evaluation, and storage of biological specimens for genetic or molecular analysis in this trial/future use {33}

Not applicable.

In this study, no biological specimens for genetic or molecular analysis will be collected.

## Statistical methods

### Statistical methods for primary and secondary outcomes {20a}

For analyses of study data, the intention-to-treat principle will be used.

For analyses of the primary outcome, the Kaplan-Meier curve, log-rank, or Wilcoxon test will be used. Standard descriptive statistics (i.e., number of available values, mean, SD, median, 25% and 75% quartiles, minimum and maximum) will be calculated for continuous variables. Categorical variables will be described by using tables of frequency. Secondary outcome measures will be compared between study groups with the *t* test or Mann-Whitney test as applicable, and the chi-square test will be used for categorical data. Differences in laboratory tests between different days of measurement will be analyzed with paired tests (paired *t* test or non-parametric Wilcoxon test). A Kaplan-Meier curve will be used to demonstrate 28-day mortality. A significance level of 5% will be used for all statistical tests. Confidence intervals will be presented as appropriate.

The data analyses will be performed after completion of the study. Interim analysis is not planned.

### Interim analyses {21b}

Not applicable.

Interim analysis is not planned.

### Methods for additional analyses (e.g., subgroup analyses) {20b}

Not applicable.

Additional analysis is not planned.

### Methods in analysis to handle protocol non-adherence and any statistical methods to handle missing data {20c}

No analysis populations are defined for this study. Protocol non-adherence will be assessed by the principal investigator case by case. Patients with major deviations from the study protocol will be excluded from the analysis.

Missing data are not planned to be imputed. However, in the event of substantial missing data for any parameter, a sensitivity analysis using any method of imputation could also be used.

### Plans to give access to the full protocol, participant level-data, and statistical code {31c}

Data sharing statement:

No later than 3 years after the collection of the 1-year postrandomization, we will deliver a completely deidentified data set to an appropriate data archive for sharing purposes.

Statistical codes will be archived in accordance with SOPs.

## Oversight and monitoring

### Composition of the coordinating center and trial steering committee {5d}

No coordinating center or a trial steering committee will be established.

### Composition of the data monitoring committee, its role, and reporting structure {21a}

Not applicable.

No data monitoring committee will be established. The study intervention is part of the standard care of critical care patients, and both modes of application of EN can be used interchangeably.

### Adverse event reporting and harms {22}

The formula of EN used in this study is approved for use in critically ill patients, and no severe adverse events relating to the components of this formula are expected.

Serious adverse events have been reported according to standard rules and standard operating procedures of the coordinating unit. All study participants will be monitored for potential adverse events associated with the administration of EN.

We expect the following adverse events to be associated with the administration of EV.

1. Tube malposition. Strategies to prevent gastric tube malposition are described in the “Intervention description {11a}” section of the protocol.
2. Gastrointestinal complications (high GRV, diarrhea, or vomiting). Management of high GRV is described in the “Intervention description {11a}” section of the protocol. These complications are considered signs of EV intolerance, which result in a decreased rate/volume of EN administered.
3. Refeeding syndrome. Strategies for prevention and management of refeeding syndrome (RFS) are part of the standard care in participating units. They include daily clinical and laboratory screening for RFS and standard medical management if RFS is diagnosed.

### Frequency and plans for auditing trial conduct {23}

Trial conduct will be audited by the Institutional Ethics Committee every 6 months during the study. The process of auditing will be independent of the investigator and sponsor.

### Plans for communicating important protocol amendments to relevant parties (e.g., trial participants, ethical committees) {25}

Any modifications to the protocol that may affect the conduct of the study, the potential benefits to the patient, or patient safety, including changes in study objectives, study design, patient population, sample sizes, study procedures, or significant administrative aspects, will require a formal amendment to the protocol. Any amendments will be agreed on and approved by the ethics committee before implementation, and the health authorities will be notified, in accordance with local regulations. The protocol amendment will be sent to all members of the study team by e-mail. All attending physicians and nurses of the study units will be acquainted with the amendment at a meeting. The invitation to the meeting will be sent by e-mail. The protocol of the study and its amendments will be available at each study unit.

## Dissemination plans {31a}

We intend to submit the results of the study to be published in a peer-reviewed international medical journal.

## Discussion

EN has been used in the treatment of critically ill patients for decades. Guidelines for its provision have been developed by several international societies [1, 11, 14] and are periodically updated. Although the body of evidence is growing, several controversies and uncertainties remain. Recent guidelines of the American Society for Parenteral and Enteral Nutrition (ASPEN) suggest that in high-risk patients and those shown to be intolerant to bolus gastric EN, the delivery of EN should be switched to continuous infusion [1]. This recommendation is based on expert consensus but is supported by very limited evidence. The studies in the analysis were small and performed on different patient populations [6, 8, 9, 15–18].

After the commencement of this study, new ESPEN guidelines on clinical nutrition in the intensive care unit were published. One recommendation is that continuous rather than bolus EN should be used. However, the authors assumed that the evidence on this issue is very limited. In a randomized clinical trial, intermittent feeding in comparison with continuous feeding has been found to result in no statistically significant difference in vomiting, abdominal distension, or diarrhea [19].. No significant differences in high GRV, abdominal distention, diarrhea, and vomiting in critically ill patients who received EF by bolus versus the continuous method have been reported [2]. A review of continuous and intermittent feeding has summarized the benefits and drawbacks of both feeding methods and found no evidence favoring either method with regard to metabolic, gastrointestinal, and organizational concerns [20].

Our protocol uses clinical signs of tolerance and GRV to determine the administration rate of EN. We use a volume of GRV > 500 mL as a sign of intolerance. In some studies, smaller volumes have been considered markers of intolerance [2]. Limitation or interruption of the administration of EN on the basis of lower GRV has been shown to lead to inadequate nutrition intake, and EN cessation is not recommended for GRV < 500 mL [1, 21, 22].

To promote EN tolerance, prokinetics will be used under the circumstances described in the “Relevant concomitant care permitted or prohibited during the trial {11d}” section of this protocol. Erythromycin or metoclopramide can be used. Both drugs are effective in decreasing feeding intolerance without increasing the rates of diarrhea and significant arrhythmias [23]. Metoclopramide has the same effectiveness regardless of the mode of administration, and the choice of a particular agent and mode of administration will be the responsibility of the attending physician [24]..

Regarding the primary outcome, the time to reach the energy target has been compared in two studies [6, 8]. After a detailed review of the study protocols, we have found that in both these studies, the increments of EN in the intermittent and continuous arm were set differently. In the case of optimal tolerance of EN, the energy target would have been reached at different time points, although the EN would have been started simultaneously in the bolus or continuous arm. Therefore, comparison of EN administration modes is problematic. In our study, we set the increments of EN so that the energy target is reached at the same time point regardless of whether EN is administered continuously or as a bolus. Therefore, both modes of EN administration are comparable regarding the primary outcome.

Aspiration of EN is a complication of EN administration. Objectively estimating aspiration is difficult. The method of coloring EN has been used in some studies. However, this method is currently not recommended for identification of aspiration because of potential adverse metabolic effects of the dye [25–27].

Aspiration of EN will be identified and noted in the medical record by nursing staff during standard airway care. This method of identification of EN aspiration is highly subjective and may be a potential source of bias.

In conclusion, we believe that this randomized trial will provide additional information regarding whether continuous or bolus feeding is associated with any differences in the tolerance and complication rate.

## Trial status

Protocol version 2.0, dated 28 May 2019. The recruitment began on 25 November 2018. One hundred patients had been randomized by 1 December 2019. The randomization of the final 300 patients is anticipated by November 2021. Completion of the follow-up will end in December 2021.

## Supplementary Information

**Additional file 1.** Ethical committee approval

## Abbreviations

ABW: Actual body weight
APACHE I: Acute Physiology and Chronic Health Evaluation
BMI: Body mass index
eCRF: Electronic case report form
EN: Enteral nutrition
ESPEN: European Society for Parenteral and Enteral Nutrition
GRV: Gastric residual volume
ICU: Intensive care unit
mNUTRIC: Modified Nutrition Risk in the Critically ill
SOFA score: Sequential Organ Failure Assessment score
VAP: Ventilator-associated pneumonia
