# Supplementary material for: Comparison of continuous versus intermittent enteral nutrition in critically ill patients (COINN): study protocol for a randomized comparative effectiveness trial
Source: Trials. 2020 Nov 23;21:955. doi: 10.1186/s13063-020-04866-2 (PMC7682133; doi:10.1186/s13063-020-04866-2)
Supplement: Supplementary file 1 — Additional file 1. Ethical committee approval [file 13063_2020_4866_MOESM1_ESM.pdf]

FAKULTNÍ NEMOCNICE BRNO

FAKULTNÍ NEMOCNICE BRNO
Jihlavská 20, 625 00 Brno
tel.: 532 232 798

MULTICENTRICKÁ ETICKÁ KOMISE
PharmDr. Šárka Kozáková, MBA
fax: 532 233 808
e-mail: etickakomise@fnbrno.cz

Ondřej Hrdý, MD
Clinic of anaesthesiology, resuscitation and intensive medicine
University Hospital Brno
Jihlavská 20
625 00 Brno

In Brno: 13Jun2018

ref. number: 02-130618

Project Title: Comparison of Continuous Versus Intermittent Enteral Nutrition in Critically Ill Patients. Effect on Energy and Protein Target Achievement, Tolerance and Incidence of Complications. Monocentric Prospective Randomised Study.

The Ethical Committee of the University Hospital Brno (FN Brno) has approved updated protocol version 2.0, dated 28May2018.

The Ethical Committee hereby declares that it operates in accordance with the relevant regulations (especially Act No. 378/2007 Sb., on pharmaceuticals, as amended, Decree No. 226/2008 Sb., on personal data protection, as amended), it follows and in the course of its work it applies in particular the principles of the Charter of Fundamental Rights and Freedoms, the Convention for the Protection of Human Rights and Dignity with regard to the application in biology and medicine, and the Declaration of Helsinki by the World Medical Association.

Yours sincerely,

ETICKÁ KOMISE
Fakultní nemocnice Brno
Jihlavská 20, 625 00 Brno

PharmDr. Šárka Kozáková, MBA
Chairman of the Ethical Committee of FN Brno
